# A Rare Association Between Prostate Cancer and Polycythemia Vera

**DOI:** 10.7759/cureus.11404

**Published:** 2020-11-09

**Authors:** Maab F Elhaj, Mohamed A Yassin

**Affiliations:** 1 Internal Medicine, Hamad Medical Corporation, Doha, QAT; 2 Hematology and Oncology, Hamad General Hospital, Doha, QAT

**Keywords:** prostate cancer, polycythemia vera, myeloproliferative neoplasms, association

## Abstract

The association of polycythemia vera (PV) and localized prostate cancer is uncommon. PV is one of the myeloproliferative neoplasms that is characterized by an increased red cell mass and uncontrolled formation of blood cells owing to an acquired mutation in Janus kinase-2. PV has numerous complications and could raise the hazard of other tumors. Here, we report an extremely rare association of localized prostate cancer in a 62-year-old male with PV. He was treated by CyberKnife surgery and completed four doses of goserelin and radiotherapy, his follow up Magnetic Resonance Imaging (MRI) four months after completion of treatment showed no recurrence, and the PV was treated with hydroxyurea 500 mg twice per day and repeated phlebotomies, when needed, to keep his hematocrit level under 45%.

## Introduction

One of the subclasses of myeloproliferative neoplasm (MPN) is the Polycythemia vera (PV), which is described as the existence of a mutated Janus kinase-2 (JAK2) exon 12 or 14 that cause panmyelosis in the bone marrow and elevated red blood cell production [1,2]. Generally, PV is an uncommon disease with a yearly occurrence of two to three for every 100,000 patients, with an average age of 65 years [2]. There has been a doubtful link between solid tumors and MPNs; nevertheless, current research has showed that the risk of acquiring solid tumors in MPN patients is twice greater than in the overall population [3]. In men, the prostate, lung, and stomach were the highest cancer risk areas; whereas, in women, the thyroid, stomach, and lungs were the most common locations [3].

We report a case of a rare association between PV and localized prostate cancer in a man with an age of 62 years. As far as we know, this is the first case report of PV, identified relying on the World Health Organization (WHO) criteria [4], to be associated with localized prostate cancer. The outcomes of the current case could assist in future identification and treatment of patients with prostate cancer and PV.

## Case presentation

A 62-year-old man presented in 2015 with a history of multiple myocardial infarctions, following percutaneous coronary intervention to the right coronary artery, left anterior descending artery, and diagonal arteries. He had a history of deep vein thrombosis and pulmonary embolism in October 2018 on lifelong anticoagulant with rivaroxaban. Also, he was diagnosed with type 2 diabetes mellitus for which oral hypoglycemic medications were prescribed. He was diagnosed with localized prostate cancer in another institution in 2018 and was treated with CyberKnife surgery and completed four doses of goserelin and radiotherapy.

The patient was followed up in National Center for Cancer Care and Research (NCCCR) in Qatar with prostate-specific antigen (PSA) level, magnetic resonance imaging (MRI) pelvis prostate, and nuclear medicine whole body fluorodeoxyglucose (FDG)-positron emission tomography-computed tomography (PET-CT).

PSA level was repeated every six months. Initially, levels were rising steadily, then they started to decline (0.16, 0.30, 0.47, 0.30 ng/mL; reference range according to the patient age: 0 to 4.5 ng/mL).

**Figure 1:**
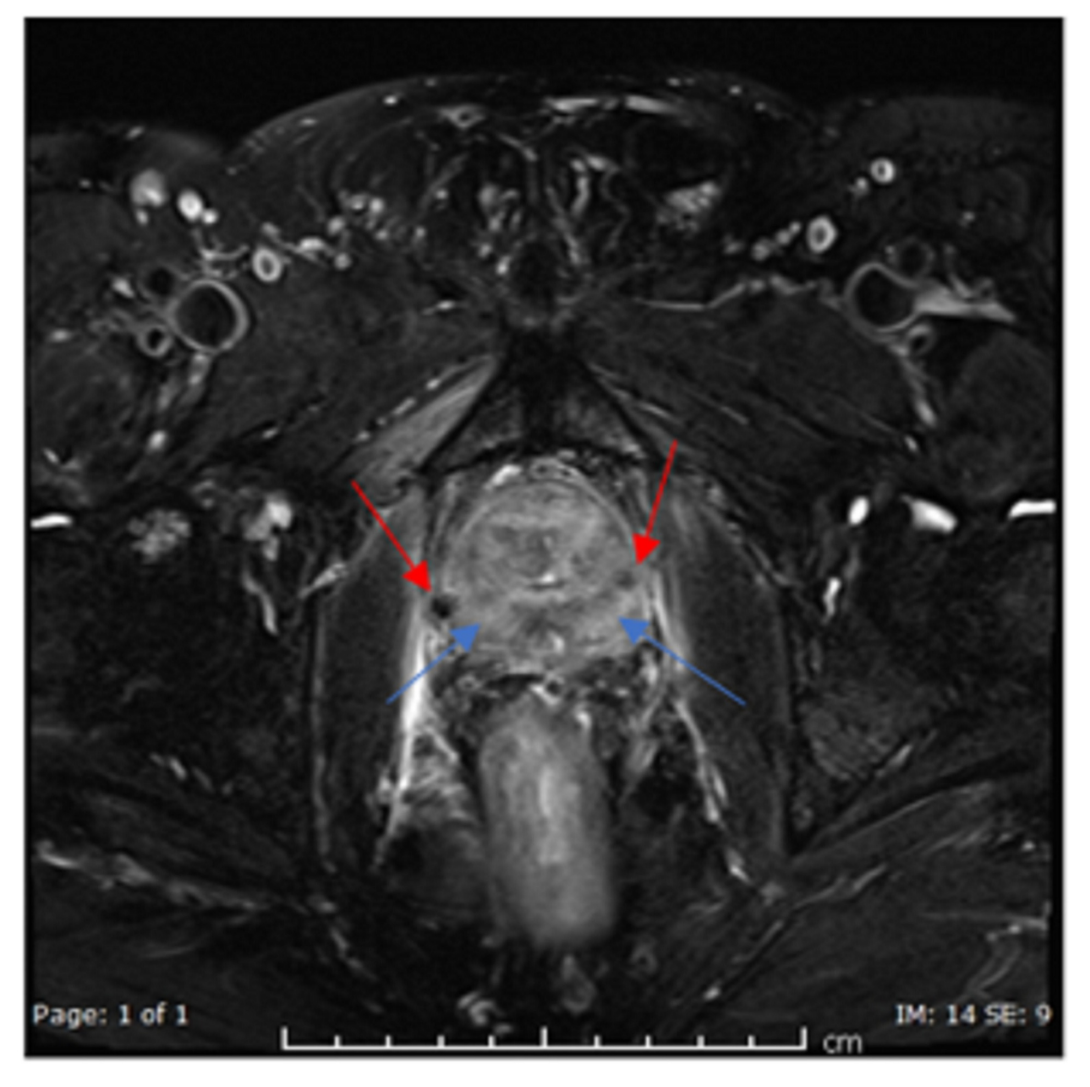
Axial T2 fat-saturated image of the prostate shows no definite masses in the peripheral zone (blue arrows). Artefacts are noted from previous intervention (red arrows).

So, follow up MRI pelvis was done to rule out local recurrence, which showed no evidence of active neoplastic disease of the pelvis (Figure 1).

**Figure 2:**
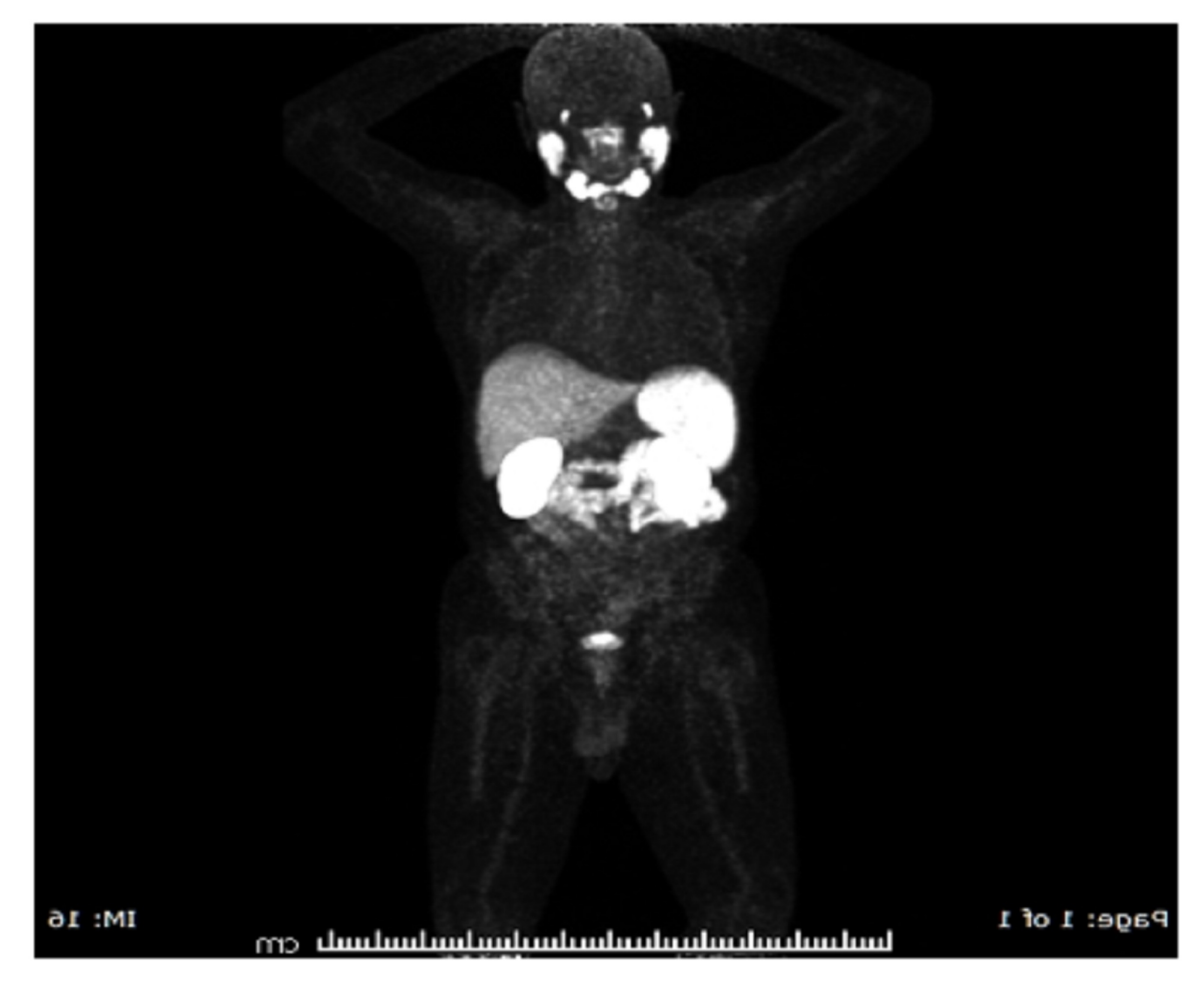
PSMA PET-CT coronal MIP image shows no detected tumor recurrence in the prostate, lymph nodes, or distant metastasis. PSMA: prostate-specific membrane antigen; PET-CT: positron emission tomography-computed tomography; MIP: maximum intensity projection

Moreover,** NM** whole-body FDG PET-CT was done, which confirmed no evidence of local, regional, or distant metastasis (Figure 2).

While hospitalized, he was found to have a high hemoglobin level of 18 g/dL (reference range: 12 to 15 g/dL) and a high hematocrit of 54 (reference range: 36 to 46). The findings of his general physical examination were normal, and his heart, chest, and abdominal examination findings were normal as well.

His JAK2 V617F mutation screen came back positive for exon 14. He had a low erythropoietin level of 1.2 (reference range: 3.7 to 36 IU/L) and had bone marrow and peripheral blood findings in accordance with MPN for which he met the 2016 WHO criteria for PV [4]. He was treated with hydroxyurea 500 mg twice per day and repeated phlebotomies, when needed, to keep his hematocrit level lower than 45%. His most recent hematocrit was 43, and his hemoglobin level was 14 g/dL.

## Discussion

Polycythemia vera is defined as a clonal disorder described by the unwarranted formation of red blood cells and is linked with JAK2 mutations (V617F or exon 12) in nearly all cases [5]. Absolute polycythemia has two kinds: primary and secondary polycythemia [6]. Usually, secondary polycythemia results from other conditions that raise the formation of erythropoietin and it is commonly linked with solid tumors [2,3], while the primary polycythemia is developed by bone marrow disorders, which mainly cause abnormal erythroid cell line production [7]. Furthermore, in rare occasions, PV can be familial when one or various MPN affect diverse kinsfolks of the same family [8]. Patients with familial MPN demonstrate similar clinical features and suffer the same complications as those with sporadic illness [8].

A pilot study was done in Qatar disclosed that familial cases of MPNs are more often acquired than defined in the literature [9], they use Clinical Exome Sequencing (CES) to sort six Qatari subjects that were supposed of clinical diagnosis of MPNs, according to the WHO 2008 diagnostic criteria for hematologic malignancies, and label variants that can probably justify the phenotypic diversity of MPNs [9].

MPNs are categorized by the WHO into numerous subclasses, and this is used for sporadic illness more willingly than familial cases, one of which is PV [9]. PV 2016 WHO criteria for diagnosis consist of one minor criterion and three main criteria [4]. The main criteria are: hematocrit >49% (men)/>48% (women), or hemoglobin >16.5 g/dL and >16.0 g/dL in men and women, respectively, or rise in red cell mass; bone marrow biopsy exhibiting hypercellularity for age with trilineage growth (panmyelosis) containing prominent granulocytic, erythroid, and megakaryocytic proliferation with different size pleomorphic, and mature megakaryocytes; and the existence of JAK2 or JAK2 exon 12 mutation. An abnormally low level of serum erythropoietin is a minor criterion. For the diagnosis of PV, either all major criteria are required or the first two major criteria in addition to an abnormally low level of serum erythropoietin are needed [4].

Moreover, thrombotic complications in patients with MPN are common: one registry study estimated that more than 30% of patients with MPN have thrombotic complications, and approximately one-third of patients with PV [10].

In a study done by Martin, she found that JAK2 V617F mutation has been associated with increased risk of thrombotic complications [11], and she concluded that prophylaxis should be introduced in high-risk patients (age >60 years and history of thrombosis) to avoid complications associated with thrombosis [11].

Prostate cancer is the most prevalent cancer diagnosis in men. However, it usually has an indolent course, it remains the third-leading cause of death among men, with greater than 160,000 new cases per year in the United States [12].

In 2010, there was an incidence of nearly 217,000 newly diagnosed prostate cancer cases and 32,000 deaths owing to prostate cancer in the United States [13]. Moreover, here in Qatar in 2010 and 2011, prostate cancer was the fourth common cancer type by incidence rate [14].

At present, the average age for prostate cancer diagnosis has reduced to 65 years, and most cases are diagnosed at local stages. Patients must decide regarding substantially various treatments, including surgery, systemic radioisotope therapy, endocrine therapy, chemotherapy, local external beam radiotherapy, denosumab and bisphosphonates are the mainstays of treatment, along with analgesics and other usual interventions [15].

Papagoras et al. published a case report on the association of dermatomyositis and neuroendocrine prostate cancer in a patient with PV [16]. Physical evaluation, electromyography, and serum creatinine phosphokinase in keeping with dermatomyositis. In parallel, the hemoglobin level was 18.5 g/dL, serum erythropoietin levels were low (within the reference range), and no JAK2 mutation was found. Therefore, they suspected paraneoplastic syndrome. The patient underwent abdominal computed tomography showing a prostate mass, fracture of L1 owing to metastasis and enlarged iliac lymph nodes. A large cell neuroendocrine prostate cancer diagnosis was made through immunohistologic examination of the needle biopsy sampling which was taken from the prostate. The patient was then referred to the oncology department for therapy [16].

Additionally, there is a reported case by Abdalhadi et al. which showed an association of PV with a benign tumor, which was a parathyroid adenoma in a woman diagnosed with PV in 2010 who fulfilled the 2008 WHO criteria [2]. The patient had hypercalcemia, and a neck ultrasound revealed regular-sized thyroid gland with heterogeneous nodules in the left, and cystic nodule in the right lobe, moreover, in the region of both parathyroid glands two hypoechoic nodes were suspected [2]. The patient was followed by an endocrinologist, who started her on cinacalcet and referred her to the surgical team for parathyroidectomy. Her PV was managed with repeated phlebotomies and hydroxyurea [2].

Moreover, there is a case report of an association between PV and prostate cancer that metastasized to the bones. According to Li et al., magnetic resonance imaging showed diffusely inhomogeneous bone marrow of the pelvis and a localized prostatic mass. The bone marrow biopsy revealed leukocytosis and erythrocytosis. The patient was managed with phlebotomy procedures electromyography two times per week and aspirin for two weeks, followed by radical prostatectomy. After the surgery, the patient was continually managed with aspirin and interferon α2b and showed no abnormalities within the one-year follow-up duration [15].

Based on the available studies and case reports, we could not reach a conclusion that there is an association between PV and prostate cancer, therefore more studies are needed to understand further if it is just a coincidence or a cause-and-effect association.

In addition, the simultaneous presence of these diseases should be well established. Moreover, the possible association between PV and prostate cancer should be well recognized by the physician in order to avoid missing or delaying the diagnosis of similar cases and to improve the quality of life of the affected patients. Finally, to our knowledge, our case could be the first case of localized prostate cancer found to have well-established PV diagnosis, based on the 2016 WHO criteria [4].

## Conclusions

We describe a rare relationship between localized prostate cancer and PV. However, the association between prostate cancer and PV has not been explored for localized prostate cancer. We want to underline this rare finding in order to increase physicians' alertness and promote further understanding of the nature of this association that would guide future therapy and improve patient quality of life.
